# Supplementary material for: Strain-specific galactose utilization by commensal E. coli mitigates Salmonella establishment in the gut
Source: PLoS Pathog. 2025 Jun 27;21(6):e1013232. doi: 10.1371/journal.ppat.1013232 (PMC12204579; doi:10.1371/journal.ppat.1013232)
Supplement: Attachment — Submitted filename: Rebuttal.docx [file ppat.1013232.s009.docx]

Here are the reviewer's comments:

Reviewer #1

In this paper, Schubert et al report the role that galactose utilization by one strain of E. coli plays on the early colonization of the gut by Salmonella. The authors identified the importance of galactose utilization in gut colonization through the screen of a transposon library. The findings obtained in the transposon screen were confirmed in competition assays between Salmonella and a few E. coli strains. These investigations come a bit short of thoroughly testing the importance of the competition for galactose by members of the microbiota on the limitation of early colonization of the gut by Salmonella.

Response: We would like to thank Referee#1 for taking the time to review our manuscript. We realized that our writing may not have been sufficiently clear, please accept our apologies. We would like to clarify that the mutant library we used was not generated through transposon mutagenesis. To gain a detailed understanding of *S.* Tm metabolism during early gut colonization in the presence of *E. coli* competitors, we utilized a rationally designed, quality-controlled pool of 35 WISH-barcoded *S.* Tm mutants, targeting key pathways for carbohydrate utilization. We have further clarified this in the manuscript and hope this will enhance the understanding of our work.

1. If E. coli limits galactose from Salmonella, then it would be predicted that the supplementation of galactose in the diet should overcome the colonization resistance seen in mice co-infected with Salmonella and E. coli 8178. The authors need to test this experimentally.

Response: We would like to thank Referee #1 for bringing the galactose supplementation experiment to our attention. To test that, we supplemented D-galactose in the drinking water of streptomycin-pretreated mice infected with a 1:1 mixture of *E. coli* 8178 and *S.* Tm (see below).

Addition of D-galactose to the drinking water (5% w/v) slightly increased the survival of *S*. Tm against *E. coli* 8178. However, this effect did not fully recapitulate the recovery of *S*. Tm when co-inoculated with the *galK*-deficient *E. coli* 8178 strain. Furthermore, it is worth noting that increasing the concentration of galactose in the drinking water (from 5% to 10% w/v) or gavaging mice with a defined amount of D-galactose (3 x 100 µL) have comparable effects without conferring a proper dose-response. Given these observations, we believe it is too early to draw definitive conclusions about the effectiveness of galactose supplementation in this context.

We believe that the relatively low impact from supplemented galactose on bacterial competition can be attributed to the host's high efficiency in absorbing galactose (Coelho et al., 2015). Additionally, it is important to note that D-galactose levels in the cecal contents of streptomycin-pretreated mice are already relatively high, typically in the single-digit millimolar range (Nguyen et al., 2024). Therefore, we do not expect that D-galactose supplementation would significantly increase cecal D-galactose levels. We have decided to not include this figure in the manuscript and have discussed the relatively limited effect of galactose supplementation and the potential reasons for this outcome. Specifically, we have added:

Line 157: *“While these observations support the idea of a galactose-rich diet as mediator for S. Tm elimination by commensal Enterobacteriaceae, it is important to note that galactose supplementation experiments have only partially, but not fully, rescued S. Tm survival against E. coli 8178. This might be attributable to the host’s relatively high efficiency in absorbing galactose or to the already sufficient levels of galactose in the caecal contents of our mice, making galactose supplementation ineffective.”*

We hope the referee will agree with this decision, though we are happy to integrate this data if requested.

2. It is confusing that one strain of E. coli competes with Salmonella for galactose, but not other strains. Nonetheless, all the strains of E. coli tested utilize galactose in vitro. The authors need to provide experimental evidence for the mechanism underlying the apparent paradox. What is the underlying cause for the apparent selectivity noticed in one strain of E. coli but not others?

Response: This is an excellent question, and we are happy to elaborate on it.

*Enterobacteriaceae* exhibit a mixotrophic lifestyle in the gut, navigating a dynamic nutritional environment that varies over time. It is presumed that *S*. Tm utilizes multiple carbohydrates, with their relative importance potentially shifting in a highly context-dependent manner (Nguyen et al., 2024; Schubert et al., 2024). Additionally, it was shown that catabolite repression enables *E. coli* to selectively and sequentially metabolize carbohydrates based on regulatory hierarchies (Okano et al., 2020). However, these hierarchies, as understood from current Systems Biology research, are primarily derived from *in vitro* studies of *E. coli* K-12. Our understanding of how these regulatory mechanisms function and drive strain-specific metabolic behaviour *in vivo* remains extremely limited.

We believe that different *E. coli* isolates possess slight variations in carbohydrate utilization hierarchies. This implies that certain isolates may have higher affinities for D-galactose or display different preferential carbohydrates than others. This could explain the differing requirements for galactose metabolism and the varying levels of competitiveness against *S.* Tm observed between *E. coli* 8178 and Z1331 *in vivo*. As rightly pointed out by the referee, this variability cannot be fully captured in simple *in vitro* growth experiments where D-galactose serves as the sole and abundant carbon source. Addressing these intricate questions from an *in vivo* perspective would require Systems Biology approaches, which fall outside the scope of our current experimental framework. However, it is important to note that despite the lack a detailed mechanistic understanding, strain-dependent competitive interactions are well-reported in the literature and have recently been observed in *Klebsiella oxytoca* spp. competing against *S.* Tm (Osbelt et al., 2024).

We agree that this critical point was lacking in the previous version of the manuscript, and we have now revised the Discussion section to include this limitation. Specifically, we have replaced:

*“Despite both E. coli 8178 and E. coli Z1331 having similar in vitro growth on galactose as the sole carbon source, only E. coli 8178 was able to exploit it and attenuate S. Tm growth in the gut. This suggests that the metabolic repertoire of a given bacterial strain alone is insufficient to predict colonization or competitive interactions in vivo”*.

with the following section:

Line 256: *“Despite both E. coli 8178 and E. coli Z1331 having similar in vitro growth on galactose as the sole carbon source, only E. coli 8178 was able to exploit it and attenuate S. Tm growth in the gut. This suggests that the metabolic repertoire of a given bacterial strain alone is insufficient to predict colonization or competitive interactions in vivo. The challenge of defining bacterial nutrient preferences in vivo stems not only from our limited understanding of the bacterial factors that drive the hierarchical consumption of carbon substrates (Okano et al., 2020) but also from the highly complex and dynamic environment that bacteria encounter within the gut microbiota (Zeng et al., 2022). Although both E. coli 8178 and Z1331 can metabolize galactose under defined in vitro conditions—where D-galactose is the sole and abundant carbon source—translating this into the gastrointestinal setting may reveal differential requirements for galactose metabolism and other growth-fuelling metabolites.”*

3. The E. coli strains, as is the case for Salmonella, are inoculated into mice in competition. Therefore, the term commensal E. coli is misleading. The idea of commensalism would be better tested in mice pre-colonized with E. coli prior to the challenge with Salmonella.
Figure 1B, the authors need to show the CFU for E. coli 8178. Moreover, what is the competitive index between Salmonella and E. coli 8178?

Response 1: Thank you for pointing out this misunderstanding. *E. coli* 8178 was originally isolated from a murine model as a commensal strain, demonstrated to be harmless to the mouse gut (Stecher et al., 2012), hence the designation as a “commensal” strain.

To avoid any confusion, we have indicated the origin of *E. coli* 8178 in the result section and have replaced:

*“E. coli 8178 a murine gut commensal strain able to outcompete S. Tm at the later stages of infection, when inflammation is established (Stecher et al., 2012).”*

with the following:

Ligne 76: *“E. coli 8178 is a commensal strain, originally isolated from the murine gut and capable of outcompeting S. Tm at the later stages of infection once inflammation is established (Stecher et al., 2012)”*

Response 2: The potential for *E. coli* 8178 to provide protection in a pre-colonization model has been tested previously (Cherrak et al., 2024). Pre-colonizing mice with *E. coli* 8178 one day prior to *S.* Tm inoculation resulted in a similar protective effect as when the strains were inoculated simultaneously (Cherrak et al., 2024 – Figure S1B-C). For the remainder of the manuscript, we opted for the co-inoculation scheme. We have updated our manuscript to include this information. Specifically, we have changed:

*“During our investigation of this phenomenon, we also discovered that E. coli 8178 mitigates S. Tm establishment during the early growth phase, consistent with recent findings (Nguyen et al., 2020; Cherrak et al., 2024a)”.*

with the following section:

Line 80: *“While investigating this phenomenon, we found out that E. coli 8178 was also able to restrict the growth of S. Tm at the early stage of infection (24 h p.i) when administered either prior to or concomitantly with S. Tm (Nguyen et al., 2020; Cherrak et al., 2024a).”*

Response 3: The population densities of *E. coli* 8178 and *E. coli* Z1331 are now shown in Figure 1D.

Response 4: Competitive indexes are commonly used to monitor and compare the growth fitness of a mutant with its isogenic wild-type (WT) strain (e.g., *E. coli* WT vs *E. coli* *galK*). Since we are performing competitive experiments using two different strains (*E. coli* and *S.* Tm), which differ in a large set of metabolic genes, we felt it would be more informative to display the loads of *S.* Tm and *E. coli* in separate graphs. This approach allows the reader to better appreciate the fitness of each strain. Following referee#1’s comment, we have now plotted the competitive index between *S*. Tm and *E. coli* (CFU *S*. Tm / CFU *E. coli*). The data is depicted in Supplementary Figure 1A.

4. What is the phenotype of the galK 8178 mutant at later times of the infection?

Response: This is an excellent question. We extended the competitive experiment between *E. coli* WT and the galK mutant to 2 days post-infection. In that scenario, we found that the fitness defect associated with the *E. coli* *galK* mutant was accentuated, indicating that *E. coli* 8178 relies on galactose for optimal growth in the gut during at least the first 2 days.

However, it is important to note that the competition between *E. coli* 8178 and *S.* Tm is, in contrast, very transient. It is visible only after 24 hours post-infection but is not aggravated between 24 and 48 hours p.i. We believe this is due to the inflammation, which has been shown to peak more severely at 48 hours p.i. in mice co-infected with *S.* Tm and *E. coli* 8178 (Cherrak et al., 2024 – Fig. 1C). Inflammation is known to induce a drastic shift in the gut metabolite landscape, modulating *S.* Tm's metabolic program. Indeed, several lines of evidence have demonstrated that *S.* Tm shifts its metabolism from monosaccharide utilization (particularly during the early stage of infection, 24h p.i.) to amino acids or SCFAs, when better electron acceptors such as nitrate and tetrathionate are available starting at 48 hours post-infection during onset of inflammation (Reviewed in: Jones et al, 2025).

Since the scope of the paper focuses on the early stage of infection (24 hours p.i.), and to avoid any confounding effects from inflammation at later stages, we conducted all our experiments at 24 hours p.i. We hope the referee will agree with this decision, though we are happy to integrate 48-hour p.i. data if requested.

5. Figure 2A is not informative. What are the genes represented by the red dots? Why were these mutations selected over other points in glycolysis, EDP and PPP?

Response: We apologize for the lack of clarity in Figure 2A. To address this, we have simplified the figure by highlighting only the names of the carbohydrates targeted in our mutant pool.

6. The competitive disadvantage of the mutants in Figure 2C seems very small (0.8 to 4 X) to be biologically relevant. Also, what does the competitive index ratio represent? Salmonella over E. coli or vice versa? In addition, data in Figure 2C cannot be interpreted correctly without results of Salmonella in SPF mice in the absence of E. coli.

Response 1: We apologize that our original explanation and presentation of the data has not been sufficiently clear. To discuss the nature of the data, we would like to focus on *galK*. The competitive disadvantage of the *galK* *S*. Tm strain (compared to WT *S*. Tm) is 0.501 in the absence of *E. coli* 8178 and 0.258 in its presence. This indicates that *E. coli* 8178 reduces the fitness of the *S.* Tm *galK* mutant by 1.94-fold, corresponding to approximately 50% reduction in its competitive fitness. These values represent median values, with p-val <0.0001 (**** using a Mann-Whitney test, n=12 animals) underscoring its statistical significance. The statistical significance of *E. coli* 8178 or 1331 effects on *S*. Tm mutant fitness is now indicated in Figure. 2C. Thereby, the reader can easily spot the significant differences.

When checking data from individual mice, this fold reduction span from 1.40 to 4.3-fold, meaning that in certain cases, the fitness of the *S.* Tm *galK* is attenuated by more than 75% in the presence of *E. coli* 8178. All individual values have been included in the Source Data file provided along with the manuscript for the reader’s review.

Whether this specific effect is biologically relevant remains an open question. We would like to bring to the referee’s attention that several studies have reported similar 2-5-fold phenotypic differences that still provide valuable insights. For example, a recent study demonstrated that the microbiota plays a crucial role in activating PPAR-γ signalling and preventing dysbiosis. This was shown, in part, through competitive experiment where the competitive index (CI) exhibited a ~3-5 fold change (Byndloss et al., 2018).

Additionally, beyond the relatively modest 2-fold difference observed in this experiment, our study demonstrates that mutating the *galK* gene in *E. coli* significantly rescues the survival of *S*. Tm by almost 10-fold, emphasizing the impact of galactose metabolism in modulating bacterial competition within the gut.

Response 2: In the original version of our manuscript, we presented the fold-change between the competitive index of each indicated *S.* Tm mutant in the absence and presence of *E. coli* 8178. We apologize for the lack of clarity. In response, we have now revised Figure 2C, which now displays only the competitive index of each *S.* Tm mutant (relative to the WT), (1) in the absence of an *E. coli* competitor (left column), (2) in the presence of *E. coli* 8178 (middle column) or (3) in the presence of E. coli Z1331 (right column). This way the reader can appreciate the reduction in the C.I of the *S*. Tm *galK* mutant - from 0.501 to 0.258 in absence and presence of *E. coli* 8178, respectively.

Response 3: We agree with the referee that merging the competitive index (C.I) of *S.* Tm alone (Supplementary Figure 2C) with Figure 2C will improve the readability of the figure. We have made this adjustment accordingly. The updated graph is shown in the revised Figure 2C.

7. Figure 2C shows similar trends between mutants in galactose utilization and the delta glpD. What is the rationale for favoring the study of the galactose mutants over the delta glpD?

Response: This is an interesting question, and we are happy to elaborate on it. Throughout our manuscript, we observed that *E. coli* 8178—but not *E. coli* Z1331—attenuates *S. Tm* growth. Consequently, we aimed to identify which *S. Tm* genes are specifically impacted by *E. coli* 8178 but not by *E. coli* Z1331, as the latter does not exhibit any significant competitive effect on the total *S*. Tm population (Figure 1C).

To address this, we focused primarily on *S.* Tm genes that displayed a differential competitive index (C.I.) in the presence of *E. coli* 8178 while remaining unaffected by *E. coli* Z1331. In the case of *glpD*, our analysis shows that the *S. Tm glpD* mutant has a fitness advantage (C.I. = 2.071) in the absence of E. coli strains, which changes in the presence of *E. coli* 8178 (C.I. = 1.046) but also in the presence of *E. coli* Z1331 (C.I. = 0.735). Since *glpD* was affected by both *E. coli* strains, it did not meet our criteria for selection.

In contrast, the *S.* Tm *galK* mutant alone has a C.I. of 0.501, which further decreases in the presence of *E. coli* 8178 (C.I. = 0.258) while remaining unchanged in the presence of *E. coli* Z1331 (C.I. = 0.51). The S. Tm *galK* strain was the only mutant that exhibited this pattern, which is why we selected it for further analysis.

Additionally, and since the reviewer is refereeing to *glpD*, we would like to highlight that the behaviour of the *S.* Tm *glpD* mutant can be explained by a phenomenon of glycerol-mediated self-intoxication, which is unrelated to *E. coli* competition and has recently been accepted for publication in a separate study (Santamaria de Souza, et al 2025, in press *Nat Comm*).

We have now updated the result section and clarified our choice to further investigate galactose metabolism. Specifically, we have replaced:

*“Given the specific effect of E. coli 8178 on the fitness of the S. Tm galactose utilization mutant, we hypothesized that galactose is the defining substrate in E. coli 8178-mediated S. Tm exclusion at this time point.”*

with the following section:

Line 163: *“**Given the competitive interaction between S. Tm and E. coli 8178 (but not E. coli Z1331, Figures 1B, C), we decided to focus on S. Tm metabolic genes that were exclusively affected by E. coli 8178 while remaining unaffected by E. coli Z1331. This highlighted galK, and broadly, galactose metabolism as a strong candidate governing E. coli 8178-mediated competition against S. Tm, which we selected for further investigation.”*

8. What is the phenotype of a galK Salmonella mutant in competition with a galK E. coli strain? If the minor defects observed are due to galactose metabolism, then colonization defect of galK Salmonella mutant is expected to disappear in competition with the galK E. coli strain. Do the small phenotypes recorded at early times translate into a competitive disadvantage at later times?

Response: This is an excellent suggestion. To address this, we triple-infected streptomycin-pretreated 129S6/SvEvTac mice with *S.* Tm WT, *S.* Tm *galK*, and an *E. coli* 8178 *galK* mutant. Interestingly, the competitive index (C.I) of the *S. Tm galK* mutant further decreased in the presence of the *E. coli* 8178 *galK* mutant. This indicates that galactose metabolism becomes even more critical for *S.* Tm when co-inoculated with a *galK*-deficient *E. coli* 8178, which differs from the referee’s initial expectations.

So far, our data have shown that *S*. Tm relies to some extent on galactose to optimally grow in the gut (C.I = 0.5), with galactose metabolism becoming even more important for *S*. Tm when co-inoculated with *E. coli* 8178 (C.I = 0.26). This indicates that the presence of *E. coli* 8178 further pushes *S*. Tm to rely on galactose utilization *in vivo*. This effect becomes more pronounced in the presence of the *galK*-deficient *E. coli* 8178 strain (C.I: ~0.01). We believe this can be explained by the fact that the *E. coli galK* mutant, due to its inability to metabolize galactose, increasingly depends on alternative carbohydrates compared to wild-type *E. coli* 8178. Consequently, this leads to a greater depletion of these alternative carbohydrate sources, further reinforcing *S.* Tm’s reliance on galactose.

The result of this experiment is depicted in Figure 3B. We have updated the result section accordingly and have added:

Line 186: *“To further confirm the capacity of E. coli 8178 to actively utilize galactose, we measured and compared S. Tm galactose-associated fitness in the presence of either the WT or galK-deficient E. coli 8178 strain. We hypothesized that co-inoculation with an E. coli strain incapable of using galactose in vivo would deplete other carbohydrates (besides galactose) even more efficiently that the WT E. coli 8178 and thereby accentuate the reliance of S. Tm on galactose. This in turn, should further exacerbate the competitive attenuation of the S. Tm galK mutant compared to WT S. Tm. Consistent with this hypothesis,* *S. Tm exhibited a significantly greater dependence on galactose when co-inoculated with a galK-deficient E. coli 8178 strain. This was shown by a S. Tm galK competitive index of approximately 0.01, which is 26 times lower than in the presence of the WT E. coli 8178 strain (C.I = 0.26) (Figure 3B, Supplementary Figure 3B).”*

9. Figure 3A, second box-and-whiskers group, are these mice infected with three strains of bacteria (i.e., E. coli WT, E. coli deficient in galk, and wild-type Salmonella)? The legend is confusing, making it difficult for the reader.

Response: Sorry for the confusion, we have modified the legend and caption accordingly:

*E. coli* 8178 (Δ*galK* + WT) refers to a co-inoculation with both the WT and *galK*-deficient *E. coli* 8178 strains.

10. Did you complement the mutant strains used in your investigations? Complementation is necessary.

Response: All WISH-barcoded *S*. Tm mutants in the library used in this study have been used and validated in a recent report (Schubert et al., 2024 – in press, in *Nature Communications*).

Complementation can be particularly challenging, when metabolic genes are involved, as their expression is often finely tuned by intricate regulatory mechanisms. When complementing in trans, we may miss these precise regulatory controls which could lead to artificial expression levels that do not accurately reflect the native context and may be detrimental in an *in vivo* setting. We have demonstrated this recently for the fine-tuning of lac operon expression by E. coli 8178 (Cherrak et al., Nature Microbiology 2024). Unfortunately, all our attempts to complement the phenotype associated with the *E. coli* 8178 *galK* strain were unsuccessful. We believe that expressing the *galK* gene *in trans* may not accurately reflect its physiological function, rendering it ineffective. As an alternative, and to rule out the possibility of secondary mutations, we tested the competitiveness of different, independently selected *galK*-deficient *E. coli* 8178 clones against *S.* Tm, —a common approach when complementation is challenging. The phenotype associated with this strain was consistently reproduced across different clones. Furthermore, whole-genome sequencing of the *E. coli* Δ*galK* strain confirmed its isogenic nature, with no non-synonymous mutations detected in genes other than *galK*. The sequencing data have been deposited at ENA ([PRJEB85163](https://www.ebi.ac.uk/ena/browser/view/PRJEB85163)).

We have acknowledged the lack of complementation in the manuscript as follows:

Line 206: *“While our complementation attempts were unsuccessful, this phenotype remained consistent across multiple galK-deficient E. coli 8178 clones. We also employed a whole genome sequencing approach that ruled out the presence of background mutations in genes other than the galK locus.”*

Minor comments:

11. Line 31, provide specifics for the WHO reference.

Response: We have provided the WHO reference as follow:

*World Health Organization. (2015). WHO estimates of the global burden of foodborne diseases: foodborne disease burden epidemiology reference group 2007-2015. World Health Organization.*

12. Line 36 and elsewhere in the manuscript, the reader can benefit from a more precise definition of the time frame in question. What do you mean by initial phase (line 36) or later stages of infection (line 62)? Hours, days, weeks?

Response: We would like to apologize for the lack of clarity. We have now added a more precise time frame which is supported by the current literature. Specifically, we have added:

Line 35: *“Research using antibiotic-pretreated specific pathogen free (SPF) C57BL/6J and gnotobiotic mouse models have identified two distinct growth phases for S. Tm in the gut (Wotzka et al., 2017). In these models,* *S. Tm establishes and grow in the intestinal lumen through monosaccharide-fuelled mixed acid fermentation during the early stage of infection (0 to 2 days post-infection) (Nguyen et al., 2020; Nguyen et al., 2024; Schubert et al., 2024; Rogers et al., 2024). Subsequently, S. Tm employs the type III secretion systems encoded within the pathogenicity island 1 (T3SS-1) and 2 (T3SS-2) to trigger inflammation, creating conditions that favour proliferation, evolution and transmission of the pathogen (Lupp et al., 2007; Stecher et al., 2007; Winter et al., 2010; Thiennimitr et al., 2011; Stecher et al., 2012; Maier et al., 2013; Faber et al., 2017; Gillis et al., 2018; Nguyen et al., 2020; Fattinger et al., 2021; Rogers et al., 2021; Nguyen et al., 2024).”*

13. Line 137, clarify the gene products of pgm, pgI and pfkA.

Response: We have indicated the gene products of pgm, pgI and pfkA as follow:

Line 145: *“Specifically, the Δpgm, ΔpgI, and ΔpfkA mutants, responsible for catalyzing the sequential conversion of D-glucose-1-phosphate to D-glucose-6-phosphate (pgm), then to D-fructose-6-phosphate (pgi), and finally to D-fructose-1,6-bisphosphate (pfkA)—exhibited a fitness disadvantage compared to the WT control. This was reflected in competitive index (C.I.) values below 1, underscoring the essential role of glycolysis under these conditions.”*

14. Line 145, where is figure 2D? Do you mean 2C?

Response: We have corrected this mistake, thank you bringing it to our attention.

15. Figure 2B is superfluous. It can be erased without undermining the clarity of the figure. Figure 3 is a logical extension of Figure 2. Merge.

Response: We apologize for the low quality of Figure 2B. We have now replaced it with a higher-quality version. We hope the referee finds this improvement helpful.

Figure 3 pertains to galactose metabolism from both the *S.* Tm and *E. coli* perspectives. Since this focus is more specific than the complete library, we have decided to separate it from Figure 2. We hope the referee finds this appropriate.

Reference:

- Byndloss, M. X., Olsan, E. E., Rivera-Chávez, F., Tiffany, C. R., Cevallos, S. A., Lokken, K. L., ... & Bäumler, A. J. (2017). Microbiota-activated PPAR-γ signaling inhibits dysbiotic Enterobacteriaceae expansion. *Science*, *357*(6351), 570-575.
- Cherrak, Y., Salazar, M. A., Yilmaz, K., Kreuzer, M., & Hardt, W. D. (2024). Commensal E. coli limits Salmonella gut invasion during inflammation by producing toxin-bound siderophores in a tonB-dependent manner. *PLoS Biology*, *22*(6), e3002616.
- Cherrak, Y., Salazar, M. A., Näpflin, N., Malfertheiner, L., Herzog, M. K. M., Schubert, C., ... & Hardt, W. D. (2024). Non-canonical start codons confer context-dependent advantages in carbohydrate utilization for commensal E. coli in the murine gut. *Nature Microbiology*, *9*(10), 2696-2709.
- Coelho, A. I., Berry, G. T., & Rubio-Gozalbo, M. E. (2015). Galactose metabolism and health. *Current Opinion in Clinical Nutrition & Metabolic Care*, *18*(4), 422-427.
- Jones, K., de Brito, C. B., & Byndloss, M. X. (2025). Metabolic tug-of-war: Microbial metabolism shapes colonization resistance against enteric pathogens. *Cell Chemical Biology*, *32*(1), 46-60.
- Nguyen, B. D., Sintsova, A., Schubert, C., Sichert, A., Scheidegger, C., Näf, J., ... & Hardt, W. D. (2024). Salmonella Typhimurium screen identifies shifts in mixed-acid fermentation during gut colonization. *Cell Host & Microbe*.
- Okano, H., Hermsen, R., Kochanowski, K. & Hwa, T. Regulation underlying hierarchical and simultaneous utilization of carbon substrates by flux sensors in Escherichia coli. *Nat Microbiol* **5**, 206-215 (2020).
- Osbelt, L., Almási, É. D. H., Wende, M., Kienesberger, S., Voltz, A., Lesker, T. R., ... & Strowig, T. (2024). Klebsiella oxytoca inhibits Salmonella infection through multiple microbiota-context-dependent mechanisms. *Nature Microbiology*, 1-20.
- Schubert, C., Nguyen, B. D., Sichert, A., Naepflin, N., Sintsova, A., Feer, L., ... & Hardt, W. D. (2024). Monosaccharides Drive Salmonella Gut Colonization in a Context-Dependent Manner. *bioRxiv*, 2024-08.
- Stecher, B., Denzler, R., Maier, L., Bernet, F., Sanders, M. J., Pickard, D. J., ... & Hardt, W. D. (2012). Gut inflammation can boost horizontal gene transfer between pathogenic and commensal Enterobacteriaceae. *Proceedings of the National Academy of Sciences*, *109*(4), 1269-1274.

— — —

Reviewer #2

The manuscript deals with clarifying mechanisms of competition in the intestinal tract between pathogenic bacteria, such as Salmonella, and pathobionts or commensals which share some niches and some biological similarities, such as E. coli. Multiple works of the so-called colonization resistance provided by some gut bacteria including E. coli against bacterial pathogens, have been presented previously by the Hardt lab and other labs that were mostly inspired by his seminal work. The group now presents a new study about sugar usage, in this case galactose, as a competitive factor between Salmonella and E. coli in the gut and mediating colonization resistance. They perform an elegant high-throughput bar-coded mutant screen in E. coli to discover and identify a galactose transporter as a source of competitive action against Salmonella enterica. They test this E. coli strain further, including diverse mutants of the same including the galactose uptake mutant, and a second gut E. coli strain, in different combinations in competitive assays in vitro; and with competitive infection assays in vivo in an antibiotic-treated, therefore Salmonella-susceptible, mouse model. They conclude that the galactose competition is a factor important in vitro and also in vivo between the tested strains, and that this trait is strain-specific for non-lab intestinal E. coli, which have a large diversity in carbohydrate uptake and utilization. The Hardt lab as suspected galactose before to serve an important function in motility-dependent niche fitness of Salmonella (2008 Cell Micro paper, doi doi: 10.1111/j.1462-5822, should be cited).

Response: We thank referee#2 for investing time in carefully reviewing our manuscript and appreciate the enthusiasm expressed. We hope our detailed point-by-point responses will be sufficient to strengthen our claim and support publication of our work. We would like to clarify that the mutant library we used was not generated through transposon mutagenesis. To gain a detailed understanding of *S.* Tm metabolism during early gut colonization in the presence of *E. coli* competitors, we utilized a rationally designed pool of 35 WISH-barcoded *S.* Tm mutants, each lacking a key metabolic enzyme. We have further clarified this in the manuscript and hope this will enhance the understanding of our work.

Response 2: We agree that a reference to our previous work on the importance of galactose as a niche for *S.* Tm growth in the gut was missing. We have now added this reference to the manuscript, which further strengthens the role of galactose in *S.* Tm luminal growth. Specifically, we have added:

Line 247: *“In line with this, galactose was shown to act as an important nutrient that promotes S. Tm growth in the gut (Stecher et al., 2008)”*

1. The study is very concise and logical, and the paper well written.
Some comments to the design - it is quite customary to test mice only with short-term Salmonella infection (here up to 24 h). The colonization seems to be stable in this set-up which could inspire the next question, whether the competitiveness also remains similar after a slightly longer duration of colonization, e.g. 48 h.

Response: This is an excellent question. We have recently investigated the impact of *E. coli* 8178 on *S*. Tm growth over a 4-day infection (Cherrak et al., 2024-Figure 1B). In this work, we found that *E. coli* 8178 was able to reduce *S*. Tm loads at different stages, mostly at 24 h p.i (early stage) and from 72 h p.i (late stage). The mechanism of *E. coli* 8178-mediated *S*. Tm competition at the 72-h p.i stage of infection was shown to involve the release of small toxins called microcins (Cherrak et al., 2024). However, the mechanism by which *E. coli* 8178 mitigates *S.* Tm growth at 24 hours was not well understood, which is the focus of our current paper. Indeed, to date, very few mechanisms of colonization resistance during the early stage of infection have been elucidated, making this an important area of investigation. This is why we chose to focus on a 24-hour infection model, which we have explained in our manuscript as follows:

Line 76: “*E. coli 8178 is a commensal strain, originally isolated from the murine gut and capable of outcompeting S. Tm at the later stages of infection once inflammation is established (Stecher et al., 2012). This competitive advantage, visible in the severely inflamed gut between 72- and 96-hours post-infection (p.i) is attributed to an interference mechanism involving the synthesis and secretion of siderophore-bound toxins (Cherrak et al., 2024a).* *While investigating this phenomenon, we found out that E. coli 8178 was also able to restrict the growth of S. Tm at the early stage of infection (24 h p.i) when administered either prior to or concomitantly with S. Tm (Nguyen et al., 2020; Cherrak et al., 2024a). To confirm this observation, we inoculated streptomycin-pretreated 129S6/SvEvTac mice harbouring a specific pathogen-free (SPF) microbiota with a 1:1 mixture of E. coli 8178 and S. Tm SL1344. Mice co-inoculated with E. coli 8178 displayed a 50-fold reduction in faecal pathogen loads at 24 hours post-infection compared to mice infected with S. Tm alone (Figure 1A, B). This significant reduction during the initial stage of S. Tm infection, which remains uncharacterized, was the focus of our investigation.”*

We would like to bring to the referee’s attention that the competition between *E. coli* 8178 and *S*. Tm at 24 h p.i is transient and disappears at 48 h p.i (Cherrak et al., Figure 1B). We believe this is due to the rise of inflammation, which peaks at 48 h p.i (Cherrak et al., 2024 – Figure 1C). Inflammation is known to induce drastic changes in the gut metabolite landscape, thus potentially modulating carbohydrate-based competition between *E. coli* and *S*. Tm. Indeed, several lines of evidence have demonstrated that *S.* Tm shifts its metabolism from monosaccharide utilization (particularly during the early stage of infection, 24h p.i.) to amino acids or SCFAs, when better electron acceptors such as nitrate and tetrathionate are available starting at 48 hours post-infection during onset of inflammation (Review: Jones et al., 2025).

Since the scope of the paper focuses on the early stage of infection (24 hours p.i.), and to avoid any confounding effects from inflammation at later stages, we conducted all our experiments at 24 hours p.i. We hope the referee will agree with this decision, though we are happy to integrate 48-hour p.i. data if requested.

2. In addition, the strain-specific aspect for E. coli also incites the question regarding possible strain-specific aspects for Salmonella as well. Another question raised by the study may be whether another type of antibiotic treatment or some remaining/reconstituted microbiota/single gut species in each case may influence the competitiveness for galactose in a certain manner.

Response: We would like to thank Referee #2 for bringing to our attention the potential effect of gut microbiota composition on *E. coli* 8178-mediated *S.* Tm competition. To investigate this, we employed a gnotobiotic mouse model with a distinct and simplified gut microbiota composition (Low Complexity Microbiota, LCM mice). The LCM microbiota is derived from the “Altered Schaedler Flora,” which is naturally permissive to *S.* Tm invasion (Dewhirst et al., 1999; Stecher et al., 2010; Maier et al., 2013). Notably, no competition was observed between *E. coli* 8178 and *S.* Tm at 24 hours post-infection, suggesting that the carbohydrate-related competition between these two intestinal bacteria is context-dependent and influenced by gut microbiota composition.

Although this observation is intriguing and highlights the importance of gut microbiota composition in shaping the behaviour of protective strains, we believe it is too preliminary to include, as we lack a clear mechanistic understanding of the underlying mechanism. Given the format of our manuscript as a short report, we prefer to keep the focus on a single, well-defined mouse model. However, if the referee requests it, we would be happy to integrate and discuss this data further.

Minor comments:
3. Fig. S3 for competitiveness could be fused with Fig. S2.

Response: Figure 3 pertains to galactose metabolism from both the *S.* Tm and *E. coli* perspectives. Since this focus is more specific than the complete library in Figure 2, we have decided to separate it from Figure 2. Therefore, we found it more appropriate to also present the related supplementary data in two separate supplementary Figures. We hope the referee agrees with this decision.

References:

- Jones, K., de Brito, C. B., & Byndloss, M. X. (2025). Metabolic tug-of-war: Microbial metabolism shapes colonization resistance against enteric pathogens. *Cell Chemical Biology*, *32*(1), 46-60.
- Stecher, B., Chaffron, S., Käppeli, R., Hapfelmeier, S., Freedrich, S., Weber, T. C., ... & Hardt, W. D. (2010). Like will to like: abundances of closely related species can predict susceptibility to intestinal colonization by pathogenic and commensal bacteria. *PLoS pathogens*, *6*(1), e1000711.
- Dewhirst, F. E., Chien, C. C., Paster, B. J., Ericson, R. L., Orcutt, R. P., Schauer, D. B., & Fox, J. G. (1999). Phylogeny of the defined murine microbiota: altered Schaedler flora. *Applied and environmental microbiology*, *65*(8), 3287-3292.
- Maier, L., Vyas, R., Cordova, C. D., Lindsay, H., Schmidt, T. S. B., Brugiroux, S., ... & Hardt, W. D. (2013). Microbiota-derived hydrogen fuels Salmonella typhimurium invasion of the gut ecosystem. *Cell host & microbe*, *14*(6), 641-651.
